# U-shaped relationship between serum phosphate and cardiovascular risk: A retrospective cohort study

**DOI:** 10.1371/journal.pone.0184774

**Published:** 2017-11-08

**Authors:** Nicholas Hayward, Andrew McGovern, Simon de Lusignan, Nicholas Cole, William Hinton, Simon Jones

**Affiliations:** 1 Department of Healthcare Management and Policy, University of Surrey, Guildford, United Kingdom; 2 Center for Healthcare Innovation and Delivery Science, Department of Population Health, New York University School of Medicine, New York, New York, United States of America; Hospital Universitario de la Princesa, SPAIN

## Abstract

**Background:**

High serum phosphate is associated with coronary artery disease in patients with normal and impaired renal function. We asked: Does the serum phosphate range provide prediction of primary cardiac events? We extracted coded primary care data for over 100,000 patients from a database of 135 primary medical practices. Patients aged between 18 and 90 years without pre-existing cardiovascular diagnoses were included from a potential sample of over 1·2 million individuals.

**Methods and findings:**

Binary logistic regression models were used to evaluate the contribution of QRISK factors and electrolytes, including serum phosphate, to cardiac outcomes at five and nine years following an initial phosphate measurement. At five-year review (n = 113,993), low serum phosphate (OR 1·75, 95%CI 1·36–2·23, p<0·001), high-normal (OR 1·50, 95%CI 1·29–1·74, p<0·001), and high serum phosphate (OR 1·74, 95%CI 1·06–2·70, p = 0·02) were long-term risk factors for primary cardiac disease events after adjusting for confounding variables. A similar pattern was seen at our nine-year review.

**Conclusions:**

The extremes of serum phosphate may confer cardiac event risk with a U-shaped trend. In particular, we raise new cardiac concerns for low serum phosphate in the general population. Also, the normal range for phosphate may require redefinition among healthy adults.

## Introduction

Cardiovascular disease is a leading cause of mortality and morbidity worldwide. Of note, high serum phosphate is an established risk factor for coronary artery disease, particularly in patients with chronic kidney disease [1]. In addition, studies have recently begun to show that high phosphate may also be a risk factor for cardiovascular disease in patients with normal renal function [2,3]. Our own previous research suggests that low serum phosphate may be associated with fewer cardiac events [4], but such trends are less well established. There is also a need to evaluate the cardiac significance of phosphate load in the context of other cardiovascular risk factors, through population based studies [4]. We aimed to investigate the predictive value of serum phosphate for new-onset cardiac disease in detail over the abnormal and normal range in a large UK primary care patient database cohort.

We hypothesized that serum phosphate influences the risk of primary cardiac disease. Therefore, we asked: Is serum phosphate a predictor of primary cardiac events including myocardial infarction, acute coronary syndrome or revascularisation procedures? Regression analyses were employed to consider the influence of serum phosphate and established QRISK factors upon primary cardiac disease [5].

## Methods

### Study design

Patient data for this study were extracted from the Royal College of General Practitioners—Research and Surveillance Centre (RCGP-RSC) database. This database houses coded primary care data from 135 General Practitioner (GP) medical practices with a total population of approximately 1·2 million patients from a mix of urban and rural UK locations. The demographics of the study population are described in Table 1. The complete protocol for the sampling and reporting of data from this database has been described previously [6].

**Table 1:** Demographic review of patients and their physiological variables retained within our regression models.

Variable	Study population		Five-year review		Nine-year review	
	n	%	n	%	n	%
All patients	113993	100	59880	100	22028	100
Age 18–40 years	17714	15.54	9289	15.51	3403	15.45
Age 41–50 years	21998	19.30	11718	19.57	4145	18.82
Age 51–60 years	25158	22.07	13547	22.62	5276	23.95
Age 61–70 years	24659	21.63	13237	22.11	4950	22.47
Age 71–80 years	17791	15.61	9469	15.81	3451	15.67
Age 81–90 years	6673	5.85	2620	4.38	803	3.65
Female	68286	59.90	36294	60.61	13335	60.54
Male	45707	40.10	23586	39.39	8693	39.46
Never smoked	47475	41.65	24680	41.22	8756	39.75
Active smoker	18724	16.43	10178	17.00	4038	18.33
Ex-smoker	45808	40.18	24193	40.40	8952	40.64
Smoking unknown	1986	1.74	829	1.38	282	1.28
No diabetes	103170	90.51	54214	90.54	19953	90.58
Diabetes	10823	9.49	5666	9.46	2075	9.42
HDL cholesterol ≤1.03 mmol/l	13861	12.16	7839	13.09	2943	13.36
HDL cholesterol 1.031–1.55 mmol/l	47123	41.34	25941	43.32	9781	44.40
HDL cholesterol >1.55 mmol/l	38463	33.74	20276	33.86	7780	35.32
HDL cholesterol unknown	14546	12.76	5824	9.73	1524	6.92
BMI ≤18.5 kg/m^2^	1518	1.33	781	1.30	295	1.34
BMI 18.6–25 kg/m^2^	22080	19.37	11460	19.14	4320	19.61
BMI 25.1–30 kg/m^2^	26388	23.15	14426	24.09	5582	25.34
BMI 30.1–40 kg/m^2^	20040	17.58	11162	18.64	4403	19.99
BMI >40 kg/m^2^	3093	2.71	1700	2.84	662	3.01
BMI unknown	40874	35.86	20351	33.99	6766	30.72
Albumin ≤35 mmol/l	4871	4.27	-	-	654	2.97
Albumin 35.1–40 mmol/l	26051	22.85	-	-	4563	20.71
Albumin 40.1–50 mmol/l	81858	71.81	-	-	16633	75.51
Albumin >50 mmol/l	1213	1.06	-	-	178	0.81
Phosphate ≤0.75 mmol/l	3545	3.11	1565	2.61	563	2.56
Phosphate 0.76–1.00 mmol/l	34098	29.91	16979	28.36	5984	27.17
Phosphate 1.01–1.25 mmol/l	58907	51.68	32139	53.67	11883	53.94
Phosphate 1.26–1.50 mmol/l	16333	14.33	8643	14.43	3371	15.30
Phosphate >1.50 mmol/l	1110	0.97	554	0.93	227	1.03

Patients aged between 18 and 90 years were included. Selected patients had no recorded myocardial infarction, acute coronary syndrome, revascularisation procedure, ischaemic stroke, peripheral vascular disease or vascular dementia prior to time zero. Time zero refers to the date of the first serum phosphate measurement. The mean phosphate measurement of up to five phosphate measurements before any cardiac event was calculated and used in our analyses. A cardiac event was defined as either myocardial infarction, acute coronary syndrome or revascularisation procedure recorded after time zero and within the study duration. This provided a composite cardiac outcome measure for this study.

### Predictors

Influential demographic and physiological variables were identified for each patient as close as possible to time zero and before any cardiac event. The demographic variables included age at time zero, gender, general practice code, ethnicity, body mass index, smoking status and UK postcode-derived deprivation score (PDDS). The physiological variables included systolic blood pressure, HDL cholesterol, LDL cholesterol, renal function (eGFR), diabetes status and blood markers HbA1c, corrected calcium, sodium, potassium, and albumin. In all cases, single measurements were used except for corrected calcium, which was recorded in a similar manner to serum phosphate with extraction of values recorded on the same dates as serum phosphate, and a mean of up to five measurements before any outcome was used in our analyses.

### Analysis

All analyses were made with the statistical package R version 3.2.3 [7]. The study variables were analysed as grouped categorical variables (Table 1). For example, serum phosphate was grouped into bands <0·75, 0·75–1·00, 1·01–1·25, 1·26–1·50, and >1·50 mmol/l. Numerical data were cleaned by removing values far outside of physiological extremes to minimise data collection errors. For each variable of interest, unrecorded or missing data were included as a separate category group in our analyses, with the exception of blood sodium, potassium, and albumin as these had ‘unknown’ categories that were too small to be included in the statistical models.

Kaplan-Meier time to outcome plots with log rank tests for each category within each variable were created to evaluate the linearity of influence on the outcome over time and over the variable range. A Cox proportional hazards multivariate model was then employed to provide a time-event analysis. However, this model narrowly failed testing of the proportional hazards assumptions (S1 Table). Thus instead, multilevel binary logistic regression models were attempted in order to account for outcome variation between different centres of primary care in our analyses. Patients were nested within their general practices using a random intercept. However, these models failed to converge and provide a reliable statistical output. This led to the creation of binary logistic regression models that included primary care practice as a confounding variable. This model was used to evaluate the contribution of our established risk factors and mean serum phosphate to cardiac outcomes at five and nine years following an initial phosphate measurement. Model selection was achieved by minimising the Bayesian information criterion (BIC) through backward stepwise elimination (step package command in R) [8]. We tested for interactions between serum phosphate and eGFR, plus serum phosphate and serum corrected calcium. The final model was checked for co-linearity of variables with none significant found. Receiver operating characteristic (ROC) curves were used to further validate the models. The analyses of this study contained no patient identifiable information and no breach of confidentiality. Ethical approval by the NHS Research Ethics Committee is needed for data requests to be considered and this approval was obtained for the present study. Other ethical considerations of studies using the RCGP-RSC database have been described previously [6].

## Results

Following data cleaning and exclusion of incomplete/likely erroneous cases, we identified 113,993 patients from the original set of patients with serum phosphate records (121,605) in the study period. This equates to 7,612 patients lost from review (6·26%). The numbers, demographic details and physiological records for the significant model variables of the whole population, the five-year review (n = 59,880) and nine-year review (n = 22,028) patients are described in Table 1.

Our analyses were based on a composite cardiac outcome measure, which included the primary cardiac events of myocardial infarction, acute coronary syndrome or revascularisation procedures combined. There were 2,369 events recorded in the total population (2·12% of patients) over the study duration. At the five-year review of eligible patients, there were 1,595 events (2·74%). During the nine-year review, there were 2,268 events (11·48%).

**Fig 1:**
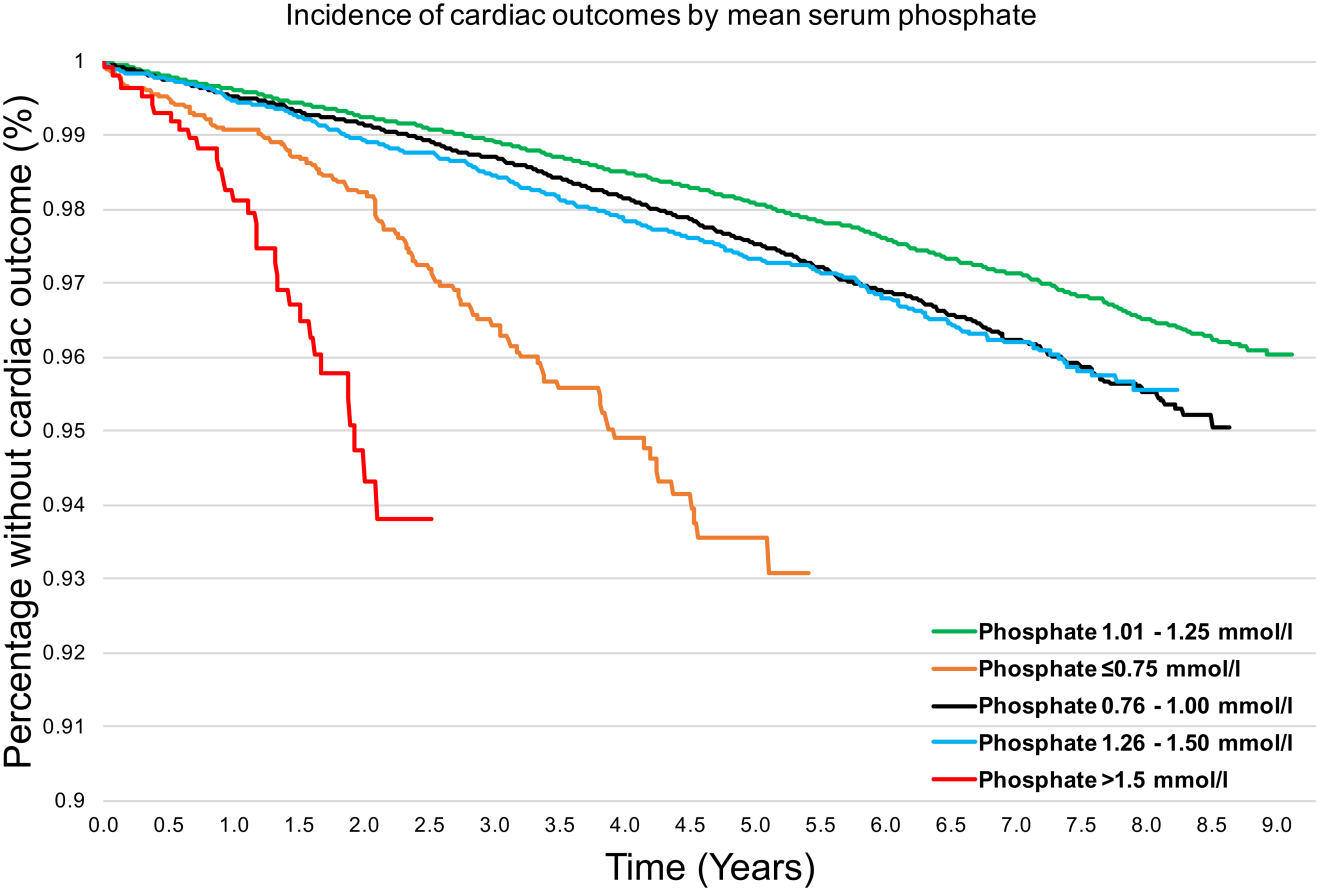
Occurrences of composite cardiac outcomes by mean phosphate group in all patients (n = 113,993) over time (years). The lowest outcome frequency was associated with mid-range normophosphatemia (green line). Conversely, serum phosphate that was high, low or at the extremes of normal range had higher outcome frequencies. Log rank test for difference between groups p<0.001.

We evaluated the frequency of cardiac outcomes across the serum phosphate range over the whole study duration for all patients (Fig 1). Fig 1 shows that the lowest outcome frequency was associated with mid-range normophosphatemia (phosphate 1·01–1·25 mmol/l, green line). Conversely, serum phosphate that was high, low or at the extremes of normal range had significantly higher frequencies of cardiac events (log rank test, p<0·001).

We asked if serum phosphate and the QRISK factors are predictors of primary cardiac events (Table 2). Table 2 shows that some established demographic factors for cardiac disease yielded significantly increased odds of primary cardiac events in this study. These included age (by decade), male gender, and active smoking at both the five-year and nine-year reviews. Diabetes during both review periods was predictive of outcome. Ethnicity, GP practice and deprivation score were lost from the stepwise analysis approach. Serum phosphate was retained within both models and demonstrated that low (OR 1·75, 95%CI 1·36–2·23, p<0·001), high-normal 1·26–1·50 mmol/l (OR 1·50, 95%CI 1·29–1·74, p<0·001) and high (OR 1·74, 95%CI 1·06–2·70, p = 0·02) mean values were associated with higher odds of primary cardiac events at the five-year review point. A similar pattern of results was observed at the nine-year review (Table 2). We tested interaction variables between phosphate and corrected calcium, as well as phosphate and eGFR, yet both interaction variables were readily lost during our stepwise regression analyses. Similarly, other individual physiological variables, including eGFR, corrected serum calcium, systolic blood pressure, serum sodium, and serum potassium had no significant impact on outcome predictions. Low HDL cholesterol (five- and nine-year) and low serum albumin (nine-year) were both associated with higher odds of a cardiac event. We found no evidence of substantial co-linearity between the variables included in each model.

**Table 2:** Results of stepwise logistic regression analyses utilising the Bayesian information criterion (BIC). Odds ratios represent the odds of a primary composite cardiac outcome at five years (n = 59880) and nine years (n = 22028) after initial serum phosphate measurement. Serum albumin was not retained during the stepwise approach at 5 years. CI = confidence interval. P values <0.05 were deemed significant. Receiver operating characteristic curve (ROC C) statistics were 0.78 and 0.79 for the five-year and nine-year reviews, respectively.

Variable	Five-year review	P value	Nine-year review	P value
	Odds ratio (95% CI)		Odds ratio (95% CI)	
Age 18–40 years	1.00 [reference]	-	1.00 [reference]	-
Age 41–50 years	4.91 (3.04–8.45)	<0.001	5.08 (3.44–7.81)	<0.001
Age 51–60 years	9.22 (5.82–15.67)	<0.001	9.12 (6.27–13.85)	<0.001
Age 61–70 years	14.35 (9.09–24.32)	<0.001	14.92 (10.28–22.62)	<0.001
Age 71–80 years	24.44 (15.49–41.45)	<0.001	27.83 (19.14–42.28)	<0.001
Age 81–90 years	43.49 (27.09–74.66)	<0.001	60.96 (40.86–94.51)	<0.001
Female	1.00 [reference]	-	1.00 [reference]	-
Male	2.00 (1.78–2.25)	<0.001	2.25 (2.02–2.50)	<0.001
Never smoked	1.00 [reference]	-	1.00 [reference]	-
Active smoker	1.64 (1.40–1.91)	<0.001	1.63 (1.42–1.87)	<0.001
Ex-smoker	1.07 (0.95–1.20)	0.28	1.09 (0.97–1.21)	0.14
Smoking unknown	2.67 (1.77–3.90)	<0.001	2.66 (1.78–3.88)	<0.001
No diabetes	1.00 [reference]	-	1.00 [reference]	-
Diabetes	1.54 (1.34–1.75)	<0.001	1.77 (1.56–2.00)	<0.001
HDL cholesterol ≤1.03 mmol/l	1.98 (1.69–2.32)	<0.001	2.12 (1.83–2.45)	<0.001
HDL cholesterol 1.031–1.55 mmol/l	1.34 (1.18–1.53)	<0.001	1.33 (1.19–1.50)	<0.001
HDL cholesterol >1.55 mmol/l	1.00 [reference]	-	1.00 [reference]	-
HDL cholesterol unknown	0.40 (0.26–0.60)	<0.001	0.41 (0.27–0.60)	<0.001
BMI ≤18.5 kg/m^2^	0.90 (0.53–1.42)	0.66	0.84 (0.53–1.28)	0.43
BMI 18.6–25 kg/m^2^	1.00 [reference]	-	1.00 [reference]	-
BMI 25.1–30 kg/m^2^	1.17 (1.01–1.35)	0.04	1.10 (0.96–1.26)	0.17
BMI 30.1–40 kg/m^2^	1.16 (0.99–1.36)	0.07	1.16 (1.00–1.34)	0.04
BMI >40 kg/m^2^	0.98 (0.67–1.38)	0.89	1.02 (0.74–1.39)	0.88
BMI unknown	0.70 (0.59–0.82)	<0.001	0.72 (0.62–0.84)	<0.001
Albumin ≤35 mmol/l	-	-	2.00 (1.61–2.46)	<0.001
Albumin 35.1–40 mmol/l	-	-	1.20 (1.08–1.34)	0.001
Albumin 40.1–50 mmol/l	-	-	1.00 [reference]	-
Albumin >50 mmol/l	-	-	0.86 (0.36–1.75)	0.70
Phosphate ≤0.75 mmol/l	1.75 (1.36–2.23)	<0.001	1.83 (1.44–2.31)	<0.001
Phosphate 0.76–1.00 mmol/l	0.99 (0.88–1.12)	0.92	1.06 (0.95–1.18)	0.31
Phosphate 1.01–1.25 mmol/l	1.00 [reference]	-	1.00 [reference]	-
Phosphate 1.26–1.50 mmol/l	1.50 (1.29–1.74)	<0.001	1.36 (1.19–1.56)	<0.001
Phosphate >1.5 mmol/l	1.74 (1.06–2.70)	0.02	1.89 (1.23–2.81)	0.003

**Fig 2:**
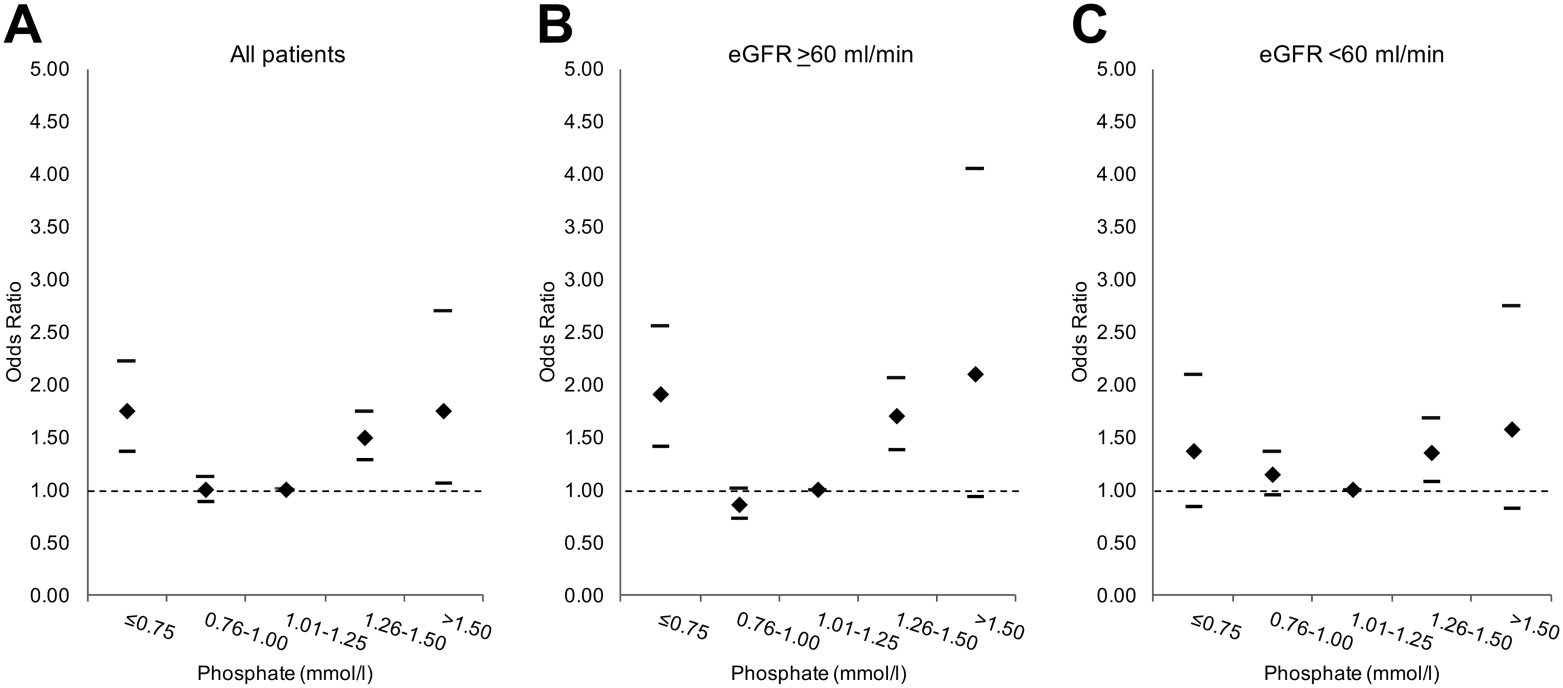
Odds ratio of composite cardiac outcome by serum phosphate at five-year review for all patients (n = 59880) (A), patients with eGFR >60 ml/min (n = 39039) (B) and patients with eGFR <60 ml/min (n = 20841) (C). The 95% confidence intervals are shown. The dotted lines identify an odds ratio of 1.00 in each graph.

We explored the variation in odds ratios over the serum phosphate range in subgroups of patients stratified by renal function within our five-year review (Fig 2). The trends in the odds ratios for cardiac events across the serum phosphate range for patients with eGFR at least 60 ml/min or below 60 ml/min were comparable as shown.

## Discussion

The present study demonstrated that low, high-normal, and high serum phosphate were associated with primary cardiac disease events in a U-shaped relationship within a general UK population. To our knowledge, this is the first study to highlight the primary cardiovascular risk significance of low serum phosphate in a large, national database cohort study. Within our analyses with interaction variables, these findings were shown to be independent of renal function and not associated with serum corrected calcium.

### Comparison with the literature

High serum phosphate is an established risk factor for cardiovascular events in patients with chronic kidney disease [1,9]. In addition, many studies have now shown that hyperphosphatemia is also relevant for the general population, irrespective of renal function, as seen in the present study. Tonelli and colleagues demonstrated that all-cause mortality and cardiovascular outcomes were associated with high and high-normal serum phosphate in patients with normal renal function [2]. Unlike in our study, the study demographic focussed on those with known coronary heart disease. A subsequent meta-analysis of similar research, which included a study mix of patients both with and without chronic kidney disease and/or established heart disease, identified a non-linear, positive relationship between serum phosphate and cardiovascular risk. Specifically, high serum phosphate was linked with an increased relative risk of cardiovascular events overall but not with a higher risk of cardiac events [3], as seen in our study. These findings were also not associated with renal function, in line with the present study. Since this meta-analysis, a recent study of a large Korean population with normal renal function demonstrated that high and high-normal serum phosphate were associated with the extent of coronary artery calcification seen by CT angiography [10]. One could speculate that this may be a mechanism behind our present findings and indeed, inorganic phosphate has long been implicated in endothelial dysfunction and atherosclerosis [9]. Coronary artery calcification has previously been linked to blood calcium load but these researchers found no such link with serum phosphate, which is contrary to the present study. This may be partly due to the different outcome measure (calcification) and smaller study sample size (n = 1088) [11]. Beyond calcification, phosphorus has many biochemical roles including signal transduction, mineral metabolism and energy exchange [3,12], thus it is difficult to further predict mechanistic explanations for its associations with cardiovascular risk.

Low serum phosphate has previously been considered as cardio-protective [2,4,13]. Our study contradicts this finding and questions the cardiac safety of hypophosphatemia, as low serum phosphate was associated with greater long-term risk of cardiac events. Although low serum phosphate has been linked to overall mortality in secondary care [14], there are only isolated case reports of cardiac events in those with re-feeding syndrome or malnutrition [15,16]. We could find no published population studies linking low serum phosphate with cardiovascular disease. Therefore, we conclude that this novel association warrants further validation in other large-scale cohort studies.

Finally, we note that many of the QRISK factors were associated with primary cardiac disease in this study, including low HDL cholesterol {7]. A novel risk factor that emerged from our 9-year review was serum albumin, even when within the low-normal range. Albumin is a carrier protein and marker of protein synthetic function [17], thus it is conceivable that its insufficiency could manifest in cardiac disease. Indeed, hypoalbuminemia has become associated with poor outcomes in heart failure patients but its precise role and primary care management is likely to be patient- and condition-specific [18].

### Implications

Our findings show that the extremes of serum phosphate are associated with increased cardiac event risk with a U-shaped trend. In particular, we raise new concerns regarding low serum phosphate in the general population. Importantly, the ‘normal range’ for serum phosphate may require redefinition among healthy adults due to the high-normal significance seen. When clinicians manage long-term cardiovascular risk, we propose that serum phosphate be carefully considered and included in their management strategies in primary care and beyond.

### Limitations of the study

The study was limited as follows. Our inclusion criteria focused on patients with serum phosphate measurements made. This potential selection bias may promote inclusion of patients with co-morbidities for which serum phosphate (or a bone profile) may require routine measurement, such as in those with bone or renal diseases. However, we excluded patients with clearly recorded, pre-existing cardiovascular disease and focused our study on novel cardiac events, which means that our relationships between serum phosphate and primary cardiac events are unlikely to be explained by any potential selection bias. Also, we note that it is beyond the scope of this study to identify the many different factors that may contribute towards physiological variation in serum phosphate.

The challenges associated with collecting routine GP data and with large observational studies are well documented [19,20]. In particular, we note that certain statistical models employed initially were unsatisfactory and thus a stepwise logistical regression approach was established thereafter (see Methods). Our study design was suited towards Cox regression analyses, although this type of model narrowly failed proportional hazards testing over the patient age range (S1 Table). Despite this, the trend in serum phosphate’s predictive value in each type of regression analysis was comparable.

### Further research

Further research is required to validate our findings and establish the potential mechanisms by which phosphate load confers cardiac outcomes. This is challenging due to the numerous metabolic and physiological roles of phosphate within the body [9,12]. It is equally important for further research to establish the causes and circumstances of blood phosphate variation in both health and disease.

### Conclusions

Low, high-normal and high serum phosphate were positively associated with long-term primary cardiac disease risk with a U-shaped trend in a large, UK-wide retrospective cohort study.

## Supporting information

S1 Table Results of the stepwise cox regression analysis utilising the Bayesian information criterion (BIC). Hazards ratios represent the odds of a primary composite cardiac outcome after initial serum phosphate measurement over the study duration for all patients (n = 113993). CI = confidence interval. P values <0.05 were deemed significant. Note proportional hazards testing results showed that our age categories failed testing of the proportional hazards assumptions, thus stepwise logistic regression analyses were employed (Table 2).
(DOCX)

S1 Checklist Document confirming study adherence to STROBE guidelines for publications. (DOC)

S1 File Research proposal protocol. (DOCX)
