# Supplementary material for: U-shaped relationship between serum phosphate and cardiovascular risk: A retrospective cohort study
Source: PLoS One. 2017 Nov 8;12(11):e0184774. doi: 10.1371/journal.pone.0184774 (PMC5695582; doi:10.1371/journal.pone.0184774)
Supplement: S1 Table — Hazards ratios represent the odds of a primary composite cardiac outcome after initial serum phosphate measurement over the study duration for all patients (n = 113993). CI = confidence interval. P values <0.05 were deemed significant. Note proportional hazards testing results showed that our age categories failed testing of the proportional hazards assumptions, thus stepwise logistic regression analyses were employed (Table 2). (DOCX) [file pone.0184774.s001.docx]

S1 Table. Results of the stepwise cox regression analysis utilising the Bayesian information criterion (BIC). Hazards ratios represent the odds of a primary composite cardiac outcome after initial serum phosphate measurement over the study duration for all patients (n=113993). CI = confidence interval. P values <0.05 were deemed significant. Note proportional hazards testing results showed that our age categories failed testing of the proportional hazards assumptions, thus stepwise logistic regression analyses were employed (Table 2).

|  |  |  |  | **Proportional Hazards Test** | | |
| --- | --- | --- | --- | --- | --- | --- |
| **Variable** | **Hazards Ratio (95% CI)** | **P value** |  | **Rho** | **Chi squared** | **P value** |
| Age 18 - 40 years | 1.00 [reference] | - |  | - | - | - |
| Age 41 - 50 years | 4.01 (2.78 - 5.80) | <0.001 |  | -0.031 | 2.314 | 0.13 |
| Age 51 - 60 years | 7.30 (5.12 - 10.41) | <0.001 |  | -0.042 | 4.217 | 0.04 |
| Age 61 - 70 years | 10.87 (7.64 - 15.46) | <0.001 |  | -0.040 | 3.893 | 0.05 |
| Age 71 - 80 years | 17.54 (12.32 - 24.95) | <0.001 |  | -0.048 | 5.542 | 0.02 |
| Age 81 - 90 years | 23.95 (16.58 - 34.59) | <0.001 |  | -0.051 | 6.318 | 0.01 |
| Female | 1.00 [reference] | - |  | - | - | - |
| Male | 1.95 (1.78 - 2.14) | <0.001 |  | 0.022 | 1.173 | 0.28 |
| Never smoked | 1.00 [reference] | - |  | - | - | - |
| Active smoker | 1.69 (1.50 - 1.91) | <0.001 |  | 0.008 | 0.163 | 0.69 |
| Ex-smoker | 1.11 (1.01 - 1.22) | 0.03 |  | -0.002 | 0.007 | 0.93 |
| Smoking unknown | 2.31 (1.66 - 3.23) | <0.001 |  | -0.034 | 2.835 | 0.09 |
| No diabetes | 1.00 [reference] | - |  | - | - | - |
| Diabetes | 1.41 (1.27 - 1.57) | <0.001 |  | -0.026 | 1.693 | 0.19 |
| HDL cholesterol ≤1.03 mmol/l | 1.88 (1.66 - 2.13) | <0.001 |  | -0.039 | 3.668 | 0.06 |
| HDL cholesterol 1.031 - 1.55 mmol/l | 1.31 (1.18 - 1.45) | <0.001 |  | -0.037 | 3.206 | 0.07 |
| HDL cholesterol >1.55 mmol/l | 1.00 [reference] | - |  | - | - | - |
| HDL cholesterol unknown | 0.32 (0.22 - 0.47) | <0.001 |  | -0.017 | 0.682 | 0.41 |
| BMI ≤18.5 kg/m^2^ | 0.85 (0.57 - 1.26) | 0.43 |  | 0.005 | 0.059 | 0.81 |
| BMI 18.6 - 25 kg/m^2^ | 1.00 [reference] | - |  | - | - | - |
| BMI 25.1 - 30 kg/m^2^ | 1.12 (0.99 - 1.25) | 0.06 |  | -0.005 | 0.061 | 0.80 |
| BMI 30.1 - 40 kg/m^2^ | 1.15 (1.01 - 1.30) | 0.03 |  | 0.007 | 0.105 | 0.75 |
| BMI >40 kg/m^2^ | 1.03 (0.78 - 1.36) | 0.84 |  | 0.015 | 0.539 | 0.46 |
| BMI unknown | 0.66 (0.58 - 0.75) | <0.001 |  | -0.009 | 0.212 | 0.65 |
| Phosphate ≤0.75 mmol/l | 1.45 (1.18 - 1.77) | <0.001 |  | 0.003 | 0.022 | 0.88 |
| Phosphate 0.76 - 1.00 mmol/l | 0.97 (0.88 - 1.07) | 0.52 |  | 0.016 | 0.592 | 0.44 |
| Phosphate 1.01 - 1.25 mmol/l | 1.00 [reference] | - |  | - | - | - |
| Phosphate 1.26 - 1.50 mmol/l | 1.37 (1.22 - 1.55) | <0.001 |  | -0.018 | 0.759 | 0.38 |
| Phosphate >1.5 mmol/l | 1.83 (1.29 - 2.58) | <0.001 |  | 0.013 | 0.400 | 0.53 |
| Global Proportional Hazards | - | - |  | N/A | 25.206 | 0.29 |
