# Supplementary material for: U-shaped relationship between serum phosphate and cardiovascular risk: A retrospective cohort study
Source: PLoS One. 2017 Nov 8;12(11):e0184774. doi: 10.1371/journal.pone.0184774 (PMC5695582; doi:10.1371/journal.pone.0184774)
Supplement: S1 File — (DOCX) [file pone.0184774.s003.docx]

**Research proposal protocol – version 6: 27/05/16**

Does serum phosphate predict cardiovascular disease outcomes?

Nick Hayward, Andy McGovern, Nicholas Cole, Will Hinton, Prof Simon de Lusignan, Prof Simon Jones

**Components**

Subjects: Retrospective cohort with serum phosphate recorded between 2005-2015

Setting: Primary Care dataset: RCGP-RSC cohort

Intervention: Serum phosphate measurements during the observation period

Outcome measure 1: Composite cardiac (atherosclerosis) cardiovascular disease outcome measure of primary myocardial infarction; acute coronary syndrome; revascularisation procedure.

Outcome measure 2: Individual cardiac (atherosclerosis) cardiovascular disease outcome measures: primary myocardial infarction; acute coronary syndrome; revascularisation procedure.

Outcome measure 3: Broader individual non-cardiac (atherosclerosis) cardiovascular disease outcome measures: ischaemic stroke; peripheral vascular disease; vascular dementia.

**Background**

Phosphate has many biochemical roles within the body. High serum phosphate predicts cardiovascular events in patients with chronic kidney disease, whilst low serum phosphate may be associated with risk reduction [1]. An association between cardiovascular events and elevated serum phosphate has also been reported in those with coronary artery disease [2], and also among the general population [3, 4, 5]. However, the precise molecular roles of phosphate in conjunction with cardiovascular risk factors are uncertain [1], and phosphate is yet to become an established marker or risk factor for cardiac events. Importantly, as vitamin D and calcium homeostasis overlap with phosphate regulation, the association of these minerals (plus parathyroid hormone) with the three outcomes will be investigated jointly. There is literature evidence that excess calcium and phosphate or their dysregulation may promote calcification during atherosclerosis [7].

The scope of this study aims to evaluate relationships between serum phosphate and the outcome measures. These outcome measures include two groups of cardiovascular events and we intend to examine them separately as follows:

Cardiovascular events due to atherosclerosis

Cardiac events (Outcomes 1 & 2) Non-cardiac events (Outcome 3)

**Hypothesis**

There is a relationship between serum phosphate concentration and the risk of cardiovascular event onset.

**Research question**

Is serum phosphate an independent predictor of new atherosclerotic cardiovascular disease events in patients stratified by renal function?

**Data source and Sample Size**

RCGP-RSC data will be used for this study. These are from a database of coded primary care data from 135 UK General Practitioner (GP) practices – total population of approximately 1,250,000 patients. A large sample size is necessary for this study due to the variability in serum phosphate concentrations and the duration of the observational period (up to ten years).

**Primary Outcome Measure**

Composite cardiac (atherosclerosis) cardiovascular disease outcome measure of primary myocardial infarction; acute coronary syndrome; revascularisation procedure.

**Secondary Outcome Measure**

Individual cardiac (atherosclerosis) cardiovascular disease outcome measures: primary myocardial infarction; acute coronary syndrome; revascularisation procedure studied separately.

**Tertiary Outcome Measure**

Individual non-cardiac cardiovascular outcome measures: ischaemic stroke; peripheral vascular disease; vascular dementia.

**Study Design**

This will be a retrospective study of the RCGP-RSC cohort. The data available were collected between April 2005 and March 2015 (Barbara to confirm). These will be analysed using Read v2 (5-bit) codes to identify patients by the inclusion and exclusion criteria. Laboratory sample data will be taken from the laboratory sample results collected by GP practices.

Inclusion: One or more serum phosphate measurements recorded before a cardiovascular event (primary myocardial infarction; acute coronary syndrome; revascularisation procedure; ischaemic stroke; peripheral vascular disease; vascular dementia), or recorded in patients without any cardiovascular event in the study period. Patients will be aged 18 years and older.

Exclusion: Patients who have already had primary myocardial infarction; acute coronary syndrome or a revascularisation procedure before any serum phosphate measurements have been recorded. Patients with pre-existing ischaemic stroke, peripheral vascular disease or vascular dementia will also be excluded.

Serum phosphate will be a linear variable or analysed further as a categorical variable by range. ‘Time zero’ will refer to the first available serum phosphate measurement of a patient. The time to any outcome measure will be recorded and associations between serum phosphate and outcomes will be analysed by cox-regression.

In patients where more than one serum phosphate measure is available, the mean average of up to the first five measurements from time zero and before any cardiovascular event will be recorded and used in this study.

Importantly, confounding factors for cardiovascular outcomes in all included patients will form part of this study. For each variable, the selected single measurement will be chosen before the outcome measure and as close to time zero as possible, subject to data availability. The exceptions are baseline eGFR and albumin:creatinine ratio – the mean of two measurements in each case will be taken.

**Confounding variables considered**

Demographics: Age (time zero); gender; ethnicity; body weight and height (to calculate body mass index); BMI; postcode-derived deprivation score (PDDS) (UK Index of Multiple Deprivation 2007); smoking status.

Co-morbidities: Cardiovascular disease (primary myocardial infarction; acute coronary syndrome; revascularisation procedure; ischaemic stroke; peripheral vascular disease; vascular dementia); HbA1c; hypertension (SBP).

**Biochemical variables required**

Serum phosphate (as detailed above); vitamin D; parathyroid hormone (PTH); sodium; potassium; cholesterol (total cholesterol, LDL cholesterol, HDL cholesterol); albumin; baseline eGFR and albumin:creatinine ratio.

**Statistical analysis**

Numerical data will be refined by excluding values beyond physiological limits. Data will be analysed using the statistical package R, with P values <0.05 being regarded as statistically significant. Expert statistical advice will be sought with the aim of using cox regression models to determine the association between serum phosphate and cardiovascular events, adjusting for the known cardiovascular risk factors. Cox proportional hazards multivariate models will also be constructed to provide a time-event analysis. A backwards stepwise elimination of variables approach will help to refine the model and evaluate the contribution of each variable towards the outcome measures.

Ethical considerations

Approval has been sought from the RCGP for the RSC database to be used for such research projects. Our study is retrospective and observational over anonymised patient data. The study involves no clinical interventions, no patient contact and no breach of confidentiality. The results of the study will be submitted for publication to a peer-reviewed journal.

**Limitations**

The study population will only include patients that have had sufficient routine blood tests taken. This may introduce bias for increased risk of confounding co-morbidities amongst this group. Serum phosphate levels are influenced acutely and chronically by many factors including disease, exercise and diet - which independently influence cardiovascular risk. We aim to control for cardiovascular risk factors based on the QRISK2 model [6].

**Intended output**

Outcome measure 1, 2 or 3 – each outcome requires its own table

|  | Outcome X | | |
| --- | --- | --- | --- |
| *Variable* | *Hazard ratio* | *95% CI* | *p value* |
| Age |  |  |  |
| Gender |  |  |  |
| Ethnicity |  |  |  |
| BMI |  |  |  |
| PDDS |  |  |  |
| Smoking |  |  |  |
| Syst. BP |  |  |  |
| HbA1c |  |  |  |
| Tot chol |  |  |  |
| LDL |  |  |  |
| HDL |  |  |  |
| Albumin |  |  |  |
| **Phosphate** |  |  |  |
| Calcium |  |  |  |
| Vit D |  |  |  |
| PTH |  |  |  |
| Sodium |  |  |  |
| Potassium |  |  |  |
| Albumin:Cr ratio |  |  |  |
| Baseline eGFR |  |  |  |

**References**

[1] McGovern AP, de Lusignan S, van Vlymen J, Liyanage H, Tomson CR, et al. (2013) Serum Phosphate as a Risk Factor for Cardiovascular Events in People with and without Chronic Kidney Disease: A Large Community Based Cohort Study. PLoS ONE 8(9): e74996. Doi: 10.1371/journal.pone.0074996

[2] Tonelli M, Sacks F, Pfeffer M, Gao Z, Curhan G (2005) Relation between serum phosphate level and cardiovascular event rate in people with coronary disease. Circulation 112: 2627–2633. doi: 10.1161/circulationaha.105.553198

[3] Dhingra R, Sullivan LM, Fox CS, Wang TJ, D'Agostino RB Sr, et al. (2007) Relations of serum phosphorus and calcium levels to the incidence of cardiovascular disease in the community. Archives of internal medicine 167: 879–885. doi: 10.1001/archinte.167.9.879

[4] Foley RN, Collins AJ, Ishani A, Kalra PA (2008) Calcium-phosphate levels and cardiovascular disease in community-dwelling adults: the Atherosclerosis Risk in Communities (ARIC) Study. Am Heart J 156: 556–563. doi: 10.1016/j.ahj.2008.05.016

[5] Abramowitz M, Muntner P, Coco M, Southern W, Lotwin I, et al. (2010) Serum alkaline phosphatase and phosphate and risk of mortality and hospitalization. Clin J Am Soc Nephrol 5: 1064–1071. doi: 10.2215/cjn.08621209

[6] <http://www.qrisk.org/>

[7] Lau et al. (2010) Phosphate and vascular calcification: Emerging role of the sodium-dependent phosphate co-transporter PiT-1
